# Mortality of Philadelphia for January, February and March, Arranged from the Record Kept at the Health Office

**Published:** 1852-06

**Authors:** Wilson Jewell


*Mortality of Philadelphia for January, February, and March, arranged from the Record kept at the Health Office.* By WILSON JEWELL, M. D.

The following tables have been carefully collated from the mortuary record kept at the Health Office, of the deaths reported to have occurred within the city proper and the several districts, making returns to the Board of Health during the first quarter of the present year, 1852; commencing on the 28th of December, 1851, and terminating on the 27th of March, ult., embracing a period of 13 weeks, or 91 days.

In classifying the causes of death, the recommendation of the American Medical Association has been followed, so far as it would serve as a guide; but in the nomenclature, the record, as found at the Health Office, has been strictly adhered to, which will account for the want of uniformity in the names of several of the diseases, and an occasional repetition.

Table No. 1 furnishes the total number of deaths for each month during the quarter, classified in the order approved by the American Medical Association.

The total number of deaths from all causes, amount to 2785. The population of those districts making returns to the Board of Health, being set down at 409,000, would give 1 death in every 181, and shows an average of 30½ deaths per day, for the quarter.

The deaths in January exceeded those in February, by 198, and in March, by 168.

The record shows an excess of deaths among males of 101.

By deducting the "still born" and the deaths from "External causes," "Old Age" and "Debility," which amount to 347, or 12 per cent. of the total, and 2438 deaths are recorded from recognizable diseases, being equal in number to 26 per day.

Out of the whole number of deaths, including the "still born," 1347, or 50 per cent., were among children under five years of age; of these, 33 pr. ct. occurred from scarlet fever, convulsions, inflammation of the lungs and bronchia, and from small pox.

Table No. 2 contains an acccurate list of the names of the several diseases found in the record, properly classified, together with the number of deaths therefrom, at fifteen distinct periods of life.

Of the deaths from disease, consumption of the lungs contributes a large share, viz., 358, equal to 14 pr. ct., and shows about four deaths daily. Small pox claims 258 deaths, varioloid 6, and variolous fever 1; in all 265, or 11 per cent. of the mortality during the quarter. It is worthy of remark, that the deaths from small pox during these three months, exceed those of any one whole year since 1804, with the exception of 1824, when the deaths for that year from small pox amounted to 325. Judging, therefore, from the present prevalence of the disease in our city, the inference is, that the mortality will be greater this year, than that of any former period. Three-fifths of the deaths from small pox have been under ten years of age.

One hundred and ninety-five deaths are set down to inflammation of the lungs, of which number, 108, or more than half, were under five years of age. Eighty-nine deaths are charged to inflammation of the bronchia, 68 of which were under five years.

Scarlet fever has furnished 126 deaths, all except six being under ten years.

Convulsions give 141 deaths; 131 occurring previous to the tenth year of life: 86 of which were under one year.

Table No. 3 presents in a summary form, the total number of deaths during the quarter, for the fifteen distinct periods of life. By this it appears that 615 or 1-5th of the whole number of deaths, took place before the termination of the 1st year of life. After this period, the highest number of deaths occurred between the ages of 20 and thirty, viz. 312.

Table No. 4 shows the highest, lowest and mean of temperature of each month during the quarter; also the amount of rain, in inches, which fell in each month during the same period.

For this table we are indebted to the record kept at the Pennsylvania Hospital by Dr. Conrad.

TABLE NO. 1.

*Deaths for the first quarter of* 1852.

| | Jany. | Feb. | Mar. | Male | Fem. | Total. |
|---|---|---|---|---|---|---|
| 1. *Endemic and Contagious Diseases.* Zymotic or Epidemic, | 244 | 231 | 201 | 342 | 334 | 676 |
| 2. *Uncertain or general seat.* Sporadic Diseases | 91 | 84 | 106 | 149 | 132 | 281 |
| 3. Of the Nervous System, | 162 | 150 | 139 | 254 | 197 | 451 |
| 4. Organs of Respiration, | 287 | 221 | 240 | 391 | 357 | 748 |
| 5. " Circulation, | 22 | 19 | 27 | 35 | 33 | 68 |
| 6. Of the Digestive Organs, | 57 | 36 | 46 | 63 | 76 | 139 |
| 7. " Urinary " | 6 | | 3 | 8 | 1 | 9 |
| 8. " Organs of Generation, | 19 | 13 | 17 | | 49 | 49 |
| 9. " Organs of Locomotion, | 6 | 5 | | 8 | 3 | 11 |
| 10. " Integumentary System, | 2 | 2 | | 1 | 3 | 4 |
| 11. Old Age, | 36 | 18 | 15 | 20 | 49 | 69 |
| 12. Of External causes, | 31 | 18 | 25 | 53 | 21 | 74 |
| Still Born, | 51 | 40 | 50 | 80 | 61 | 141 |
| Unknown, | 23 | 22 | 20 | 39 | 26 | 65 |
| | 1037 | 859 | 889 | 1443 | 1342 | 2785 |

TABLE No. 2.

1. *Endemic and Contagious Diseases.—Zymotic or Epidemic.*

| | Male. | Female. | Under 1 yr. | 1 to 2. | 2 to 5. | 5 to 10. | 10 to 15. | 15 to 20. | 20 to 30. | 30 to 40. | 40 to 50. | 50 to 60. | 60 to 70. | 70 to 80. | 80 to 90. | 90 to 100. | 100 to 110. | Total. |
|---|---|---|---|---|---|---|---|---|---|---|---|---|---|---|---|---|---|---|
| Cholera Infantum . . | 2 | 1 | 3 | 0 | 0 | 0 | 0 | 0 | 0 | 0 | 0 | 0 | 0 | 0 | 0 | 0 | 0 | 3 |
| Croup . . . | 33 | 28 | 13 | 9 | 32 | 7 | 0 | 0 | 0 | 0 | 0 | 0 | 0 | 0 | 0 | 0 | 0 | 61 |
| Diarrhœa . . . | 13 | 13 | 8 | 4 | 2 | 1 | 0 | 1 | 4 | 1 | 1 | 2 | 1 | 1 | 0 | 0 | 0 | 26 |
| Dysentery . . . | 17 | 17 | 5 | 3 | 5 | 4 | 1 | 2 | 1 | 5 | 2 | 4 | 2 | 0 | 0 | 0 | 0 | 34 |
| Erysipelas . . . | 19 | 15 | 15 | 1 | 0 | 1 | 0 | 0 | 6 | 6 | 3 | 4 | 3 | 2 | 1 | 2 | 0 | 44 |
| Fever . . . . | 3 | 4 | 1 | 2 | 1 | 1 | 0 | 0 | 1 | 0 | 0 | 0 | 0 | 0 | 0 | 1 | 0 | 7 |
| " Congestive . . | 1 | 3 | 0 | 0 | 0 | 0 | 1 | 0 | 0 | 0 | 0 | 2 | 1 | 0 | 0 | 0 | 0 | 4 |
| " Bilious . . . | 0 | 1 | 0 | 0 | 1 | 0 | 0 | 0 | 0 | 0 | 0 | 0 | 0 | 0 | 0 | 0 | 0 | 1 |
| " Intermittent . . | 1 | 0 | 0 | 0 | 0 | 0 | 0 | 0 | 0 | 1 | 0 | 0 | 0 | 0 | 0 | 0 | 0 | 1 |
| " Remittent . . | 3 | 3 | 0 | 0 | 0 | 0 | 0 | 0 | 0 | 0 | 3 | 0 | 1 | 2 | 0 | 0 | 0 | 6 |
| " Scarlet . . . | 55 | 71 | 14 | 30 | 43 | 32 | 1 | 3 | 3 | 0 | 0 | 0 | 0 | 0 | 0 | 0 | 0 | 126 |
| " Typhus . . | 16 | 12 | 1 | 0 | 0 | 1 | 0 | 2 | 9 | 6 | 3 | 4 | 2 | 0 | 0 | 0 | 0 | 28 |
| " Typhoid . . | 9 | 11 | 0 | 2 | 2 | 0 | 0 | 3 | 8 | 1 | 3 | 0 | 0 | 1 | 0 | 0 | 0 | 20 |
| " Variolous . . | 0 | 1 | 1 | 0 | 0 | 0 | 0 | 0 | 1 | 0 | 0 | 0 | 0 | 0 | 0 | 0 | 0 | 1 |
| Hooping Cough . . | 4 | 3 | 3 | 1 | 1 | 1 | 0 | 0 | 1 | 0 | 0 | 0 | 0 | 0 | 0 | 0 | 0 | 7 |
| Influenza . . . | 0 | 3 | 1 | 0 | 0 | 0 | 0 | 0 | 0 | 0 | 0 | 0 | 1 | 1 | 0 | 0 | 0 | 3 |
| Measles . . . . | 21 | 17 | 5 | 15 | 13 | 4 | 0 | 0 | 0 | 1 | 0 | 0 | 0 | 0 | 0 | 0 | 0 | 38 |
| Small Pox . . . | 142 | 116 | 55 | 29 | 53 | 27 | 6 | 16 | 33 | 15 | 14 | 7 | 0 | 3 | 0 | 0 | 0 | 258 |
| Syphilis . . . | 1 | 1 | 1 | 0 | 0 | 0 | 0 | 0 | 1 | 0 | 0 | 0 | 0 | 0 | 0 | 0 | 0 | 2 |
| Varioloid . . . | 2 | 4 | 1 | 3 | 1 | 0 | 0 | 1 | 0 | 0 | 0 | 0 | 0 | 0 | 0 | 0 | 0 | 6 |
| | 342 | 334 | 127 | 99 | 154 | 79 | 9 | 28 | 67 | 36 | 29 | 23 | 11 | 10 | 1 | 3 | 0 | 676 |

2. *Uncertain or General Seat.—Sporadic Diseases.*

| | Male. | Female. | Under 1 yr. | 1 to 2. | 2 to 5. | 5 to 10. | 10 to 15. | 15 to 20. | 20 to 30. | 30 to 40. | 40 to 50. | 50 to 60. | 60 to 70. | 70 to 80. | 80 to 90. | 90 to 100. | 100 to 110. | Total. |
|---|---|---|---|---|---|---|---|---|---|---|---|---|---|---|---|---|---|---|
| Abscess . . . | 1 | 1 | 0 | 2 | 0 | 0 | 0 | 0 | 0 | 0 | 0 | 0 | 0 | 0 | 0 | 0 | 0 | 2 |
| " Psoas . . . | 1 | 0 | 0 | 0 | 0 | 0 | 0 | 0 | 1 | 0 | 0 | 0 | 0 | 0 | 0 | 0 | 0 | 1 |
| " Throat . . . | 1 | 0 | 0 | 0 | 1 | 0 | 0 | 0 | 0 | 0 | 0 | 0 | 0 | 0 | 0 | 0 | 0 | 1 |
| Cachexia . . . | 2 | 0 | 0 | 0 | 0 | 0 | 0 | 0 | 1 | 0 | 1 | 0 | 0 | 0 | 0 | 0 | 0 | 2 |
| Cancer . . . | 1 | 2 | 0 | 0 | 0 | 0 | 0 | 0 | 0 | 0 | 1 | 1 | 1 | 0 | 0 | 0 | 0 | 3 |
| " Breast . . . | 0 | 4 | 0 | 0 | 0 | 0 | 0 | 0 | 0 | 0 | 1 | 1 | 1 | 1 | 0 | 0 | 0 | 4 |
| " Sore Mouth . . | 1 | 0 | 1 | 0 | 0 | 0 | 0 | 0 | 0 | 0 | 0 | 0 | 0 | 0 | 0 | 0 | 0 | 1 |
| " Cheek and Tongue | 1 | 0 | 0 | 0 | 0 | 0 | 0 | 0 | 0 | 0 | 0 | 0 | 1 | 0 | 0 | 0 | 0 | 1 |
| Congestion . . . | 1 | 1 | 1 | 0 | 0 | 0 | 0 | 0 | 0 | 0 | 1 | 0 | 0 | 0 | 0 | 0 | 0 | 2 |
| Cyanosis . . . | 5 | 4 | 9 | 0 | 0 | 0 | 0 | 0 | 0 | 0 | 0 | 0 | 0 | 0 | 0 | 0 | 0 | 9 |
| Debility . . . | 36 | 27 | 37 | 4 | 0 | 0 | 1 | 1 | 2 | 3 | 4 | 9 | 1 | 1 | 0 | 0 | 0 | 63 |
| Dropsy . . . . | 12 | 14 | 0 | 3 | 4 | 2 | 0 | 1 | 1 | 4 | 3 | 3 | 3 | 2 | 0 | 0 | 0 | 26 |
| Gangrene of Foot . . | 1 | 0 | 0 | 0 | 0 | 0 | 0 | 0 | 0 | 0 | 0 | 0 | 0 | 1 | 0 | 0 | 0 | 1 |
| Gout . . . . . | 3 | 0 | 0 | 0 | 0 | 0 | 0 | 0 | 0 | 0 | 0 | 2 | 1 | 0 | 0 | 0 | 0 | 3 |
| Hemorrhage . . . | 5 | 7 | 4 | 1 | 0 | 1 | 0 | 0 | 3 | 3 | 0 | 0 | 0 | 0 | 0 | 0 | 0 | 12 |
| Inanition . . . | 15 | 14 | 25 | 2 | 0 | 0 | 0 | 0 | 0 | 0 | 1 | 0 | 0 | 1 | 0 | 0 | 0 | 29 |
| Inflammation . . | 1 | 2 | 1 | 0 | 0 | 0 | 0 | 0 | 1 | 1 | 0 | 0 | 0 | 0 | 0 | 0 | 0 | 3 |
| Inflam. Parotid Gland . | 0 | 1 | 1 | 0 | 0 | 0 | 0 | 0 | 0 | 0 | 0 | 0 | 0 | 0 | 0 | 0 | 0 | 1 |
| Malformation . . | 5 | 2 | 7 | 0 | 0 | 0 | 0 | 0 | 0 | 0 | 0 | 0 | 0 | 0 | 0 | 0 | 0 | 7 |
| Marasmus . . . | 44 | 38 | 42 | 14 | 13 | 1 | 1 | 1 | 2 | 1 | 3 | 2 | 1 | 0 | 1 | 0 | 0 | 82 |
| Mortification . . | 2 | 2 | 1 | 1 | 0 | 0 | 0 | 0 | 1 | 0 | 0 | 0 | 0 | 0 | 1 | 0 | 0 | 4 |
| Mortification of Foot . | 0 | 1 | 0 | 0 | 0 | 0 | 0 | 0 | 0 | 0 | 0 | 0 | 0 | 0 | 1 | 0 | 0 | 1 |
| Scrofula . . . | 7 | 3 | 1 | 1 | 4 | 1 | 0 | 0 | 1 | 0 | 0 | 2 | 0 | 0 | 0 | 0 | 0 | 10 |
| Tabes Mesenterica . | 2 | 5 | 2 | 5 | 0 | 0 | 0 | 0 | 0 | 0 | 0 | 0 | 0 | 0 | 0 | 0 | 0 | 7 |
| Ulceration . . . | 1 | 1 | 0 | 0 | 0 | 0 | 0 | 1 | 0 | 0 | 0 | 0 | 0 | 0 | 1 | 0 | 0 | 2 |
| " Throat . . | 1 | 2 | 1 | 0 | 0 | 0 | 0 | 0 | 1 | 1 | 0 | 0 | 0 | 0 | 0 | 0 | 0 | 3 |
| " Neck . . . | 0 | 1 | 0 | 0 | 0 | 0 | 0 | 0 | 0 | 0 | 1 | 0 | 0 | 0 | 1 | 0 | 0 | 1 |
| | 149 | 132 | 133 | 33 | 22 | 5 | 2 | 4 | 14 | 13 | 16 | 20 | 9 | 6 | 4 | 0 | 0 | 281 |

3. *Of the Nervous System.*

| | Male. | Female. | Under 1 yr. | 1 to 2. | 2 to 5. | 5 to 10. | 10 to 15. | 15 to 20. | 20 to 30. | 30 to 40. | 40 to 50. | 50 to 60. | 60 to 70. | 70 to 80. | 80 to 90. | 90 to 100. | 100 to 110. | Total. |
|---|---|---|---|---|---|---|---|---|---|---|---|---|---|---|---|---|---|---|
| Abscess of Brain . . | 1 | 0 | 0 | 0 | 0 | 0 | 0 | 0 | 0 | 1 | 0 | 0 | 0 | 0 | 0 | 0 | 0 | 1 |
| Apoplexy . . . | 13 | 11 | 2 | 0 | 0 | 0 | 0 | 0 | 0 | 2 | 7 | 2 | 1 | 6 | 4 | 0 | 0 | 24 |
| Chorea . . . . | 1 | 2 | 1 | 0 | 0 | 0 | 1 | 1 | 0 | 0 | 0 | 0 | 0 | 0 | 0 | 0 | 0 | 3 |
| Convulsions . . . | 76 | 65 | 86 | 19 | 18 | 8 | 1 | 3 | 0 | 2 | 1 | 3 | 0 | 0 | 0 | 0 | 0 | 141 |
| " Puerperal . | 0 | 7 | 0 | 0 | 0 | 0 | 1 | 0 | 3 | 3 | 0 | 0 | 0 | 0 | 0 | 0 | 0 | 7 |
| Cerebral Fever . . | 1 | 1 | 0 | 1 | 0 | 1 | 0 | 0 | 0 | 0 | 0 | 0 | 0 | 0 | 0 | 0 | 0 | 2 |
| Concussion of Brain . | 1 | 2 | 1 | 0 | 0 | 0 | 0 | 0 | 0 | 0 | 0 | 0 | 2 | 0 | 0 | 0 | 0 | 3 |
| Congestion " . | 18 | 7 | 4 | 1 | 3 | 1 | 0 | 0 | 4 | 4 | 1 | 2 | 2 | 2 | 0 | 1 | 0 | 25 |
| Disease " . | 14 | 17 | 9 | 7 | 5 | 3 | 2 | 0 | 2 | 0 | 0 | 2 | 1 | 0 | 0 | 0 | 0 | 31 |
| Dropsy " . | 34 | 31 | 21 | 24 | 16 | 4 | 0 | 0 | 0 | 0 | 0 | 0 | 0 | 0 | 0 | 0 | 0 | 65 |
| Effusion " . | 13 | 8 | 8 | 3 | 6 | 2 | 1 | 0 | 0 | 1 | 0 | 0 | 0 | 0 | 0 | 0 | 0 | 21 |
| Epilepsy . . . | 3 | 1 | 1 | 0 | 0 | 0 | 0 | 0 | 0 | 1 | 1 | 0 | 0 | 0 | 1 | 0 | 0 | 4 |
| Inflammation of Brain . | 42 | 27 | 15 | 18 | 12 | 7 | 3 | 3 | 4 | 3 | 0 | 3 | 1 | 0 | 0 | 0 | 0 | 69 |
| Mania a Potu . . | 13 | 2 | 0 | 0 | 0 | 0 | 0 | 0 | 1 | 2 | 5 | 2 | 5 | 0 | 0 | 0 | 0 | 15 |
| Palsy . . . . | 20 | 14 | 0 | 0 | 1 | 0 | 0 | 0 | 1 | 3 | 2 | 5 | 9 | 10 | 2 | 1 | 0 | 34 |
| Softening of Brain . . | 0 | 2 | 0 | 0 | 0 | 0 | 0 | 0 | 0 | 0 | 0 | 1 | 0 | 1 | 0 | 0 | 0 | 2 |
| Spasm of Glottis . . | 1 | 0 | 1 | 0 | 0 | 0 | 0 | 0 | 0 | 0 | 0 | 0 | 0 | 0 | 0 | 0 | 0 | 1 |
| Thymic Asthma . . | 1 | 0 | 0 | 0 | 1 | 0 | 0 | 0 | 0 | 0 | 0 | 0 | 0 | 0 | 0 | 0 | 0 | 1 |
| Tetanus . . . | 1 | 0 | 0 | 0 | 0 | 0 | 0 | 0 | 0 | 0 | 0 | 0 | 1 | 0 | 0 | 0 | 0 | 1 |
| Tympanitis . . . | 1 | 0 | 1 | 0 | 0 | 0 | 0 | 0 | 0 | 0 | 0 | 0 | 0 | 0 | 0 | 0 | 0 | 1 |
| | 254 | 197 | 150 | 73 | 62 | 26 | 9 | 7 | 15 | 22 | 17 | 20 | 22 | 19 | 7 | 2 | 0 | 451 |

### 4. *Organs of Respiration.*

| | Male. | Female. | Under 1 yr. | 1 to 2. | 2 to 5. | 5 to 10. | 10 to 15. | 15 to 20. | 20 to 30. | 30 to 40. | 40 to 50. | 50 to 60. | 60 to 70. | 70 to 80. | 80 to 90. | 90 to 100. | 100 to 110. | Total. |
|---|---|---|---|---|---|---|---|---|---|---|---|---|---|---|---|---|---|---|
| Abscess of Lungs . . | 6 | 1 | 0 | 1 | 1 | 1 | 0 | 0 | 1 | 2 | 0 | 1 | 0 | 0 | 0 | 0 | 0 | 7 |
| Asthma . . . | 1 | 2 | 0 | 0 | 1 | 0 | 0 | 0 | 1 | 0 | 0 | 0 | 1 | 0 | 0 | 0 | 0 | 3 |
| Congestion of Lungs . | 14 | 12 | 9 | 3 | 3 | 0 | 1 | 0 | 2 | 0 | 1 | 1 | 3 | 1 | 1 | 1 | 0 | 26 |
| Consumption " . | 181 | 177 | 7 | 10 | 3 | 7 | 2 | 17 | 109 | 86 | 56 | 29 | 22 | 8 | 2 | 0 | 0 | 358 |
| " Laryngeal . | 2 | 1 | 0 | 0 | 0 | 0 | 0 | 0 | 0 | 0 | 1 | 2 | 0 | 0 | 0 | 0 | 0 | 3 |
| Disease of Lungs . . | 2 | 4 | 2 | 0 | 0 | 0 | 0 | 0 | 1 | 1 | 0 | 0 | 2 | 0 | 0 | 0 | 0 | 6 |
| Dropsy of Chest . . | 6 | 4 | 0 | 0 | 2 | 0 | 0 | 0 | 2 | 1 | 1 | 1 | 2 | 1 | 0 | 0 | 0 | 10 |
| Hemorrhage from Lungs | 4 | 3 | 0 | 0 | 0 | 0 | 0 | 1 | 1 | 0 | 1 | 1 | 1 | 2 | 0 | 0 | 0 | 7 |
| Hepatization of " . | 0 | 1 | 0 | 0 | 0 | 0 | 0 | 0 | 0 | 0 | 0 | 0 | 0 | 1 | 0 | 0 | 0 | 1 |
| Inflammation of " . | 105 | 90 | 55 | 24 | 29 | 7 | 3 | 5 | 19 | 10 | 12 | 12 | 6 | 11 | 1 | 1 | 0 | 195 |
| " Chest . . | 3 | 5 | 4 | 2 | 0 | 0 | 0 | 0 | 0 | 0 | 0 | 0 | 1 | 1 | 0 | 0 | 0 | 8 |
| " Pleura . . | 3 | 1 | 0 | 0 | 0 | 0 | 0 | 1 | 1 | 0 | 1 | 0 | 0 | 1 | 0 | 0 | 0 | 4 |
| " Larynx . . | 4 | 3 | 0 | 1 | 1 | 0 | 0 | 0 | 2 | 2 | 0 | 1 | 0 | 0 | 0 | 0 | 0 | 7 |
| " Throat . . | 3 | 1 | 0 | 0 | 2 | 0 | 0 | 0 | 1 | 1 | 0 | 0 | 0 | 0 | 0 | 0 | 0 | 4 |
| " Bronchia . | 46 | 42 | 44 | 16 | 8 | 2 | 0 | 0 | 2 | 3 | 1 | 3 | 4 | 2 | 3 | 0 | 0 | 88 |
| Disease of Chest . . | 2 | 1 | 0 | 1 | 0 | 0 | 1 | 0 | 0 | 0 | 1 | 0 | 0 | 0 | 0 | 0 | 0 | 3 |
| Effusion " . . | 0 | 2 | 0 | 0 | 0 | 1 | 0 | 0 | 0 | 0 | 0 | 0 | 0 | 1 | 0 | 0 | 0 | 2 |
| Tuberculosis . . | 6 | 6 | 1 | 1 | 1 | 0 | 2 | 2 | 1 | 1 | 0 | 2 | 1 | 0 | 0 | 0 | 0 | 12 |
| Ulceration of Larynx . | 1 | 0 | 0 | 0 | 0 | 1 | 0 | 0 | 0 | 0 | 0 | 0 | 0 | 0 | 0 | 0 | 0 | 1 |
| Coryza . . . . | 0 | 1 | 1 | 0 | 0 | 0 | 0 | 0 | 0 | 0 | 0 | 0 | 0 | 0 | 0 | 0 | 0 | 1 |
| Empyema . . . | 1 | 0 | 0 | 0 | 0 | 0 | 0 | 1 | 0 | 0 | 0 | 0 | 0 | 0 | 0 | 0 | 0 | 1 |
| Effusion of Pleura . . | 1 | 0 | 0 | 0 | 0 | 0 | 0 | 0 | 0 | 1 | 0 | 0 | 0 | 0 | 0 | 0 | 0 | 1 |
| | 391 | 357 | 123 | 59 | 51 | 19 | 9 | 27 | 143 | 108 | 75 | 53 | 43 | 29 | 7 | 2 | 0 | 748 |

### 5. *Of the Organs of Circulation.*

| | Male. | Female. | Under 1 yr. | 1to 2. | 2 to 5. | 5 to 10. | 10 to 15. | 15 to 20. | 20 to 30. | 30 to 40. | 40 to 50. | 50 to 60. | 60 to 70. | 70 to 80. | 80 to 90. | 90 to 100. | 100 to 110. | Total. |
|---|---|---|---|---|---|---|---|---|---|---|---|---|---|---|---|---|---|---|
| Aneurism of Aorta . . | 0 | 1 | 0 | 0 | 0 | 1 | 0 | 0 | 0 | 0 | 0 | 0 | 0 | 0 | 0 | 0 | 0 | 1 |
| Angina Pectoris , . | 2 | 1 | 0 | 0 | 0 | 0 | 0 | 0 | 0 | 0 | 0 | 1 | 2 | 0 | 0 | 0 | 0 | 3 |
| Anæmia . . . | 3 | 0 | 1 | 0 | 0 | 0 | 0 | 0 | 0 | 0 | 0 | 1 | 1 | 0 | 0 | 0 | 0 | 3 |
| Disease of Heart . . | 22 | 16 | 5 | 0 | 2 | 0 | 0 | 2 | 7 | 5 | 3 | 7 | 3 | 4 | 0 | 0 | 0 | 38 |
| Dropsy " . . | 0 | 1 | 0 | 0 | 0 | 1 | 0 | 0 | 0 | 0 | 0 | 0 | 0 | 0 | 0 | 0 | 0 | 1 |
| Effusion " . . | 0 | 2 | 0 | 0 | 0 | 1 | 0 | 0 | 0 | 1 | 0 | 0 | 0 | 0 | 0 | 0 | 0 | 2 |
| Enlargement " . . | 3 | 7 | 0 | 0 | 0 | 0 | 0 | 1 | 0 | 4 | 0 | 3 | 2 | 0 | 0 | 0 | 0 | 10 |
| Inflammation " . . | 4 | 3 | 1 | 0 | 1 | 0 | 1 | 0 | 2 | 0 | 0 | 1 | 1 | 0 | 0 | 0 | 0 | 7 |
| " Vein . . | 1 | 1 | 0 | 0 | 0 | 0 | 0 | 0 | 1 | 0 | 0 | 1 | 0 | 0 | 0 | 0 | 0 | 2 |
| Malformation of Heart . | 1 | 0 | 1 | 0 | 0 | 0 | 0 | 0 | 0 | 0 | 0 | 0 | 0 | 0 | 0 | 0 | 0 | 1 |
| | 36 | 32 | 8 | 0 | 3 | 3 | 1 | 3 | 10 | 10 | 3 | 14 | 9 | 4 | 0 | 0 | 0 | 68 |

6. *Of the Digestive Organs.*

| | Male. | Female. | Under 1 yr. | 1 to 2. | 2 to 5. | 5 to 10. | 10 to 15. | 15 to 20. | 20 to 30. | 30 to 40. | 40 to 50. | 50 to 60. | 60 to 70. | 70 to 80. | 80 to 90. | 90 to 100. | 100 to 110. | Total. |
|---|---|---|---|---|---|---|---|---|---|---|---|---|---|---|---|---|---|---|
| Abscess of Spleen . . | 0 | 1 | 1 | 0 | 0 | 0 | 0 | 0 | 0 | 0 | 0 | 0 | 0 | 0 | 0 | 0 | 0 | 1 |
| " Liver . . . | 1 | 0 | 0 | 0 | 0 | 0 | 0 | 0 | 1 | 0 | 0 | 0 | 0 | 0 | 0 | 0 | 0 | 1 |
| Abdominal Dropsy . | 5 | 2 | 0 | 0 | 0 | 1 | 0 | 0 | 1 | 1 | 1 | 1 | 0 | 1 | 1 | 0 | 0 | 7 |
| Cancer of Stomach . | 3 | 1 | 0 | 0 | 0 | 0 | 0 | 0 | 0 | 1 | 0 | 2 | 1 | 0 | 0 | 0 | 0 | 4 |
| " Pylorus . | 1 | 0 | 0 | 0 | 0 | 0 | 0 | 0 | 0 | 1 | 0 | 0 | 0 | 0 | 0 | 0 | 0 | 1 |
| Colic . . . . . | 0 | 4 | 2 | 0 | 0 | 0 | 0 | 0 | 0 | 0 | 1 | 1 | 0 | 0 | 0 | 0 | 0 | 4 |
| Congestion of Liver . | 3 | 2 | 4 | 0 | 0 | 0 | 0 | 0 | 0 | 0 | 0 | 1 | 0 | 0 | 0 | 0 | 0 | 5 |
| Disease of stom.& bowels | 4 | 3 | 2 | 1 | 0 | 0 | 0 | 0 | 1 | 1 | 0 | 1 | 1 | 0 | 0 | 0 | 0 | 7 |
| " Stomach . | 0 | 2 | 0 | 0 | 0 | 0 | 0 | 0 | 0 | 0 | 0 | 1 | 1 | 0 | 0 | 0 | 0 | 2 |
| " Liver . . | 3 | 1 | 1 | 0 | 0 | 0 | 0 | 0 | 0 | 1 | 1 | 0 | 1 | 0 | 0 | 0 | 0 | 4 |
| Fatty Degen. of Liver . | 1 | 0 | 0 | 0 | 0 | 0 | 0 | 0 | 0 | 0 | 0 | 1 | 0 | 0 | 0 | 0 | 0 | 1 |
| Gastric Fever . . | 1 | 0 | 0 | 1 | 0 | 0 | 0 | 0 | 0 | 0 | 0 | 0 | 0 | 0 | 0 | 0 | 0 | 1 |
| Hemor'ge from Bowels . | 1 | 1 | 1 | 0 | 0 | 0 | 0 | 0 | 0 | 0 | 0 | 1 | 0 | 0 | 0 | 0 | 0 | 2 |
| Hernia . . . . | 2 | 2 | 1 | 0 | 0 | 0 | 0 | 0 | 0 | 1 | 1 | 0 | 0 | 1 | 0 | 0 | 0 | 4 |
| Cancrum Oris . . | 1 | 0 | 0 | 1 | 0 | 0 | 0 | 0 | 0 | 0 | 0 | 0 | 0 | 0 | 0 | 0 | 0 | 1 |
| Inflam. Stom & Bowels . | 17 | 27 | 20 | 4 | 1 | 1 | 0 | 1 | 5 | 5 | 2 | 2 | 1 | 2 | 0 | 0 | 0 | 44 |
| " Liver . . . | 11 | 3 | 2 | 1 | 0 | 0 | 0 | 0 | 3 | 2 | 1 | 1 | 1 | 1 | 2 | 0 | 0 | 14 |
| " Peritoneum . . | 3 | 20 | 0 | 0 | 0 | 0 | 0 | 1 | 12 | 3 | 2 | 3 | 0 | 2 | 0 | 0 | 0 | 23 |
| Intussusception . . | 1 | 0 | 1 | 0 | 0 | 0 | 0 | 0 | 0 | 0 | 0 | 0 | 0 | 0 | 0 | 0 | 0 | 1 |
| Jaundice . . . | 0 | 2 | 1 | 0 | 1 | 0 | 0 | 0 | 0 | 0 | 0 | 0 | 0 | 0 | 0 | 0 | 0 | 2 |
| Mortification of Bowels | 1 | 0 | 0 | 0 | 0 | 0 | 0 | 0 | 0 | 0 | 1 | 0 | 0 | 0 | 0 | 0 | 0 | 1 |
| Perforation " . | 1 | 1 | 0 | 0 | 0 | 0 | 0 | 0 | 1 | 0 | 1 | 0 | 0 | 0 | 0 | 0 | 0 | 2 |
| Schirrus of Pancreas . | 0 | 1 | 0 | 0 | 0 | 0 | 0 | 0 | 0 | 1 | 0 | 0 | 0 | 0 | 0 | 0 | 0 | 1 |
| Ulcerat. Mouth & Throat | 0 | 1 | 1 | 0 | 0 | 0 | 0 | 0 | 0 | 0 | 0 | 0 | 0 | 0 | 0 | 0 | 0 | 1 |
| " Stomach . . | 1 | 1 | 0 | 1 | 0 | 0 | 0 | 0 | 0 | 0 | 0 | 0 | 0 | 1 | 0 | 0 | 0 | 2 |
| " Bowels . . | 1 | 1 | 0 | 0 | 1 | 0 | 0 | 0 | 1 | 0 | 0 | 0 | 0 | 0 | 0 | 0 | 0 | 2 |
| Worms . . . | 1 | 0 | 0 | 0 | 1 | 0 | 0 | 0 | 0 | 0 | 0 | 0 | 0 | 0 | 0 | 0 | 0 | 1 |
| | 63 | 76 | 37 | 9 | 4 | 2 | 0 | 2 | 25 | 17 | 11 | 15 | 6 | 8 | 3 | 0 | 0 | 139 |

7. *Of the Urinary Organs.*

| | Male. | Female. | Under 1 yr. | 1 to 2. | 2 to 5. | 5 to 10. | 10 to 15. | 15 to 20. | 20 to 30. | 30 to 40. | 40 to 50. | 50 to 60. | 60 to 70. | 70 to 80. | 80 to 90 | 90 to 100. | 100 to 110. | Total. |
|---|---|---|---|---|---|---|---|---|---|---|---|---|---|---|---|---|---|---|
| Disease of Kidneys . | 1 | 0 | 0 | 0 | 0 | 0 | 0 | 0 | 0 | 0 | 0 | 1 | 0 | 0 | 0 | 0 | 0 | 1 |
| „ Bladder . | 1 | 0 | 0 | 0 | 0 | 0 | 0 | 0 | 0 | 0 | 0 | 0 | 0 | 1 | 0 | 0 | 0 | 1 |
| „ Urinary organs | 0 | 1 | 0 | 0 | 0 | 0 | 0 | 0 | 0 | 0 | 0 | 0 | 0 | 0 | 1 | 0 | 0 | 1 |
| Inflam. of Bladder . | 4 | 0 | 0 | 0 | 0 | 0 | 0 | 0 | 2 | 0 | 0 | 1 | 0 | 0 | 1 | 0 | 0 | 4 |
| „ Kidneys . | 2 | 0 | 1 | 0 | 0 | 0 | 0 | 1 | 0 | 0 | 0 | 0 | 0 | 0 | 0 | 0 | 0 | 2 |
| | 8 | 1 | 1 | 0 | 0 | 0 | 0 | 1 | 2 | 0 | 0 | 2 | 0 | 1 | 2 | 0 | 0 | 9 |

8. *Of the Organs of Generation.*

| | Male. | Female. | Under 1 yr. | 1 to 2. | 2 to 5. | 5 to 10. | 10 to 15. | 15 to 20. | 20 to 30. | 30 to 40. | 40 to 50. | 50 to 60. | 60 to 70. | 70 to 80. | 80 to 90. | 90 to 100. | 100 to 110. | Total. |
|---|---|---|---|---|---|---|---|---|---|---|---|---|---|---|---|---|---|---|
| Cancer of Uterus . . | 0 | 5 | 0 | 0 | 0 | 0 | 0 | 0 | 0 | 1 | 1 | 3 | 0 | 0 | 0 | 0 | 0 | 5 |
| Childbed . . . | 0 | 1 | 0 | 0 | 0 | 0 | 0 | 0 | 0 | 1 | 0 | 0 | 0 | 0 | 0 | 0 | 0 | 1 |
| Hemorr'ge from Uterus . | 0 | 3 | 0 | 0 | 0 | 0 | 0 | 0 | 0 | 2 | 1 | 0 | 0 | 0 | 0 | 0 | 0 | 3 |
| Inflammation of " . | 0 | 5 | 0 | 0 | 0 | 0 | 0 | 0 | 4 | 1 | 0 | 0 | 0 | 0 | 0 | 0 | 0 | 5 |
| Menorrhagia . . | 0 | 1 | 0 | 0 | 0 | 0 | 0 | 0 | 0 | 0 | 1 | 0 | 0 | 0 | 0 | 0 | 0 | 1 |
| Puerperal Fever . . | 0 | 27 | 0 | 0 | 0 | 0 | 0 | 2 | 10 | 12 | 3 | 0 | 0 | 0 | 0 | 0 | 0 | 27 |
| Ovarian Tumors . . | 0 | 2 | 0 | 0 | 0 | 0 | 0 | 0 | 0 | 0 | 1 | 0 | 1 | 0 | 0 | 0 | 0 | 2 |
| " Dropsy . . | 0 | 1 | 0 | 0 | 0 | 0 | 0 | 0 | 1 | 0 | 0 | 0 | 0 | 0 | 0 | 0 | 0 | 1 |
| Chlorosis . . . | 0 | 1 | 0 | 0 | 0 | 0 | 0 | 0 | 0 | 1 | 0 | 0 | 0 | 0 | 0 | 0 | 0 | 1 |
| Phlegmasia Dolens . | 0 | 1 | 0 | 0 | 0 | 0 | 0 | 0 | 1 | 0 | 0 | 0 | 0 | 0 | 0 | 0 | 0 | 1 |
| Puerperal Phlebitis . | 0 | 1 | 0 | 0 | 0 | 0 | 0 | 0 | 0 | 1 | 0 | 0 | 0 | 0 | 0 | 0 | 0 | 1 |
| Rupture of Uterus . . | 0 | 1 | 0 | 0 | 0 | 0 | 0 | 0 | 0 | 1 | 0 | 0 | 0 | 0 | 0 | 0 | 0 | 1 |
| | 0 | 49 | 0 | 0 | 0 | 0 | 0 | 2 | 16 | 20 | 7 | 3 | 1 | 0 | 0 | 0 | 0 | 49 |

9. *Of the Organs of Locomotion.*

| | Male. | Female. | Under 1 yr. | 1 to 2. | 2 to 5. | 5 to 10. | 10 to 15. | 15 to 20. | 20 to 30. | 30 to 40. | 40 to 50. | 50 to 60. | 60 to 70. | 70 to 80. | 80 to 90. | 90 to 100. | 100 to 110. | Total. |
|---|---|---|---|---|---|---|---|---|---|---|---|---|---|---|---|---|---|---|
| Caries of Spine . . | 2 | 0 | 0 | 1 | 0 | 0 | 0 | 0 | 0 | 1 | 0 | 0 | 0 | 0 | 0 | 0 | 0 | 2 |
| Hip Disease . . . | 1 | 0 | 0 | 0 | 0 | 1 | 0 | 0 | 0 | 0 | 0 | 0 | 0 | 0 | 0 | 0 | 0 | 1 |
| Malformation of Spine . | 1 | 0 | 1 | 0 | 0 | 0 | 0 | 0 | 0 | 0 | 0 | 0 | 0 | 0 | 0 | 0 | 0 | 1 |
| Spinal Irritation . . | 0 | 1 | 0 | 0 | 1 | 0 | 0 | 0 | 0 | 0 | 0 | 0 | 0 | 0 | 0 | 0 | 0 | 1 |
| Spina Bifida . . . | 1 | 1 | 2 | 0 | 0 | 0 | 0 | 0 | 0 | 0 | 0 | 0 | 0 | 0 | 0 | 0 | 0 | 2 |
| Rheumatism . . . | 3 | 1 | 0 | 0 | 0 | 1 | 0 | 2 | 0 | 0 | 0 | 0 | 0 | 0 | 1 | 0 | 0 | 4 |
| | 8 | 3 | 3 | 1 | 1 | 2 | 0 | 2 | 0 | 1 | 0 | 0 | 0 | 0 | 1 | 0 | 0 | 11 |

10. *Of the Integumentary System.*

| | Male. | Female. | Under 1 yr. | 1 to 2. | 2 to 5. | 5 to 10. | 10 to 15. | 15 to 20. | 20 to 30. | 30 to 40. | 40 to 50. | 50 to 60. | 60 to 70. | 70 to 80. | 80 to 90. | 90 to 100. | 100 to 110. | Total. |
|---|---|---|---|---|---|---|---|---|---|---|---|---|---|---|---|---|---|---|
| Anthrax . . . . | 0 | 1 | 0 | 0 | 0 | 0 | 0 | 0 | 0 | 0 | 1 | 0 | 0 | 0 | 0 | 0 | 0 | 1 |
| Purpura Hemorrhagica . | 0 | 1 | 0 | 0 | 1 | 0 | 0 | 0 | 0 | 0 | 0 | 0 | 0 | 0 | 0 | 0 | 0 | 1 |
| Ulcer . . . . . | 0 | 1 | 0 | 0 | 0 | 0 | 0 | 0 | 0 | 0 | 0 | 0 | 0 | 0 | 1 | 0 | 0 | 1 |
| Ulceration of Cheek . | 1 | 0 | 0 | 1 | 0 | 0 | 0 | 0 | 0 | 0 | 0 | 0 | 0 | 0 | 0 | 0 | 0 | 1 |
| | 1 | 3 | 0 | 1 | 1 | 0 | 0 | 0 | 0 | 0 | 1 | 0 | 0 | 0 | 1 | 0 | 0 | 4 |

| | | | | | | | | | | | | | | | | | | |
|---|---|---|---|---|---|---|---|---|---|---|---|---|---|---|---|---|---|---|
| 11. *Old Age* . . | 20 | 49 | 0 | 0 | 0 | 0 | 0 | 0 | 0 | 0 | 0 | 0 | 6 | 23 | 33 | 5 | 2 | 69 |

12. *From External Causes.*

| | Male. | Female. | Under 1 yr. | 1 to 2. | 2 to 5. | 5 to 10. | 10 to 15. | 15 to 20. | 20 to 30. | 30 to 40. | 40 to 50. | 50 to 60. | 60 to 70. | 70 to 80. | 89 to 90. | 90 to 100. | 100 to 110. | Total. |
|---|---|---|---|---|---|---|---|---|---|---|---|---|---|---|---|---|---|---|
| Asphyxia . . . | 2 | 5 | 4 | 0 | 0 | 0 | 0 | 0 | 0 | 0 | 3 | 0 | 0 | 0 | 0 | 0 | 0 | 7 |
| Burns and Scalds . . | 7 | 3 | 2 | 0 | 3 | 1 | 0 | 0 | 0 | 0 | 2 | 1 | 0 | 1 | 0 | 0 | 0 | 10 |
| Casualties . . . | 19 | 3 | 2 | 0 | 3 | 0 | 1 | 0 | 8 | 1 | 5 | 1 | 0 | 1 | 0 | 0 | 0 | 22 |
| Drowned . . . | 4 | 0 | 0 | 0 | 0 | 0 | 0 | 0 | 0 | 2 | 2 | 0 | 0 | 0 | 0 | 0 | 0 | 4 |
| Fracture of Thigh . . | 1 | 0 | 0 | 0 | 0 | 0 | 0 | 0 | 0 | 0 | 0 | 0 | 0 | 1 | 0 | 0 | 0 | 1 |
| Fracture of Skull . . | 1 | 0 | 0 | 0 | 0 | 0 | 0 | 0 | 1 | 0 | 0 | 0 | 0 | 0 | 0 | 0 | 0 | 1 |
| Intemperance . . | 10 | 4 | 0 | 0 | 0 | 0 | 0 | 0 | 1 | 4 | 4 | 3 | 2 | 0 | 0 | 0 | 0 | 14 |
| Neglect . . . | 0 | 1 | 0 | 0 | 0 | 0 | 0 | 0 | 1 | 0 | 0 | 0 | 0 | 0 | 0 | 0 | 0 | 1 |
| Poisoning . . . | 1 | 1 | 0 | 0 | 0 | 0 | 0 | 0 | 1 | 1 | 0 | 0 | 0 | 0 | 0 | 0 | 0 | 2 |
| Suffocation . . . | 2 | 2 | 0 | 1 | 0 | 1 | 0 | 0 | 1 | 0 | 0 | 0 | 0 | 1 | 0 | 0 | 0 | 4 |
| Suicide . . . . | 3 | 2 | 0 | 0 | 0 | 0 | 0 | 1 | 0 | 2 | 0 | 1 | 1 | 0 | 0 | 0 | 0 | 5 |
| Violence . . . | 3 | 0 | 0 | 0 | 0 | 0 | 0 | 1 | 2 | 0 | 0 | 0 | 0 | 0 | 0 | 0 | 0 | 3 |
| | 53 | 21 | 8 | 1 | 6 | 2 | 1 | 2 | 15 | 10 | 16 | 6 | 3 | 4 | 0 | 0 | 0 | 74 |
| Unknown . . . | 39 | 26 | 25 | 4 | 7 | 2 | 0 | 0 | 5 | 3 | 7 | 7 | 3 | 1 | 1 | 0 | 0 | 65 |
| Still born . . . | 80 | 61 | 0 | 0 | 0 | 0 | 0 | 0 | 0 | 0 | 0 | 0 | 0 | 0 | 0 | 0 | 0 | 141 |

TABLE NO. 3.

*Deaths during the quarter at fifteen distinct periods of life.*

| | |
|---|---|
| Under 1 year, . . . . . . | 615 |
| 1 to 2 . . . . . . . | 280 |
| 2 to 5 . . . . . . . | 311 |
| 5 to 10 . . . . . . . | 140 |
| 10 to 15 . . . . . . . | 31 |
| 15 to 20 . . . . . . . | 78 |
| 20 to 30 . . . . . . . | 312 |
| 30 to 40 . . . . . . . | 240 |
| 40 to 50 . . . . . . . | 182 |
| 50 to 60 . . . . . . . | 163 |
| 60 to 70 . . . . . . . | 113 |
| 70 to 80 . . . . . . . | 105 |
| 80 to 90 . . . . . . . | 60 |
| 90 to 100 . . . . . . . | 12 |
| 100 to 110 . . . . . . . | 2 |
| | 2644 |
| Still Born . . . . . . . | 141 |
| Total, | 2785 |

Of the deaths recorded during the quarter, 261 were people of color; 258 were from the almshouse and 16 were from the country, as follows:

| | Blacks. | Almshouse. | Country. |
|---|---|---|---|
| January . . . . | 90 | 89 | |
| February . . . . . | 83 | 84 | 5 |
| March . . . . | 88 | 85 | 11 |
| Total, | 261 | 258 | 16 |

TABLE NO. 4.

*Meteorological Summary.*

| | TEMPERATURE. | | | SNOW AND RAIN. |
|---|---|---|---|---|
| | Highest. | Lowest. | Mean. | Inches. |
| January | 33.23° | 21.77° | 27.05° | 2.011 |
| February | 39.96 | 28.15 | 34.05 | 2.810 |
| March | 49.90 | 33.61 | 40.75 | 4.270 |

